# Effects of evidence-based early mobilization on prognostic outcomes in older patients with acute ischemic stroke

**DOI:** 10.3389/fpubh.2026.1774045

**Published:** 2026-03-16

**Authors:** Yang-Xi Mao, Yuan Zhang, Ya-Hong Wen, Chen Hu

**Affiliations:** 1 Department of Neurology, Chongqing Traditional Chinese Medicine Hospital, Chongqing, China; 2 School of Nursing, Chongqing University of Chinese Medicine, Chongqing, China

**Keywords:** acute ischemic stroke, early mobilization, older adults, evidence-based rehabilitation, modified Rankin Scale, National Institutes of Health Stroke Scale

## Abstract

**Background:**

Early mobilization is a key component of stroke-unit rehabilitation, yet evidence regarding structured, protocolized early mobilization in older adults with acute ischemic stroke remains limited. This study aimed to evaluate whether an evidence-based early mobilization (EBEM) program, added to conventional rehabilitation, is associated with improved short-term outcomes in older patients with acute ischemic stroke.

**Methods:**

This retrospective cohort study included consecutive patients aged ≥60 years with imaging-confirmed acute ischemic stroke admitted between January 2023 and January 2025. Patients treated during 2023 received conventional rehabilitation (control group), whereas those treated during 2024 received EBEM in addition to conventional rehabilitation (observation group). The primary outcome was favorable functional status at discharge, defined as modified Rankin Scale (mRS) score 0–2. Secondary outcomes included neurological recovery measured by the National Institutes of Health Stroke Scale (NIHSS), functional and motor outcomes, gait performance, length of stay, and in-hospital complications. Multivariable logistic regression, subgroup analyses, and propensity score–based sensitivity analyses were performed.

**Results:**

A total of 177 patients were analyzed (control *n* = 86; EBEM *n* = 91). Favorable functional outcome occurred more frequently in the EBEM group (54.9% vs. 34.9%; *p* = 0.011). EBEM was independently associated with favorable outcome after adjustment (adjusted odds ratio 3.57, 95% confidence interval 1.61–7.93; *p* = 0.002). Greater neurological improvement was observed (NIHSS change 4.66 ± 1.27 vs. 3.67 ± 1.31; *p* < 0.001), along with superior Barthel Index, Fugl–Meyer Assessment motor score, Berg Balance Scale, walking distance, and walking speed (all *p* < 0.001). EBEM was associated with shorter hospitalization (*p* = 0.010), fewer intensive care unit transfers (*p* = 0.045), and lower rates of pneumonia (9.9% vs. 20.9%; *p* = 0.041), venous thromboembolism (2.2% vs. 10.5%; *p* = 0.023), and pressure injury (1.1% vs. 7.0%; *p* = 0.045). Sensitivity analyses yielded consistent findings.

**Conclusion:**

Evidence-based early mobilization added to conventional rehabilitation may be associated with improved short-term functional and neurological outcomes and fewer immobility-related complications in older patients with acute ischemic stroke.

## 1 Introduction

Acute ischemic stroke (AIS) remains a leading cause of death and long-term disability worldwide, and the burden is disproportionately borne by older adults, who frequently present with higher comorbidity load, reduced physiological reserve, and greater vulnerability to immobility-related complications. Contemporary stroke rehabilitation frameworks emphasize early, coordinated inpatient rehabilitation to prevent secondary complications and to facilitate recovery of activities of daily living (ADL), motor control, and balance, while acknowledging the need for individualized dosing and careful safety monitoring in the acute phase. Recent guideline updates and evidence summaries highlight structured multidisciplinary rehabilitation as a core element of stroke unit care and recommend timely assessment and initiation of therapy tailored to patient tolerance and goals (1, 2). Early mobilization, broadly defined as graded out-of-bed activity initiated during the acute hospitalization, has been increasingly incorporated into clinical pathways. Over the past 3 years, growing evidence suggests that appropriately timed and protocolized early mobilization may improve early functional recovery and reduce immobility-related adverse events, although heterogeneity persists with respect to optimal initiation window, intensity, and progression criteria (3, 4). Studies focusing on very early or structured early mobilization approaches report potential benefits in functional outcomes and shortened hospitalization when physiologic stability and dose titration are prioritized (5). Implementation research further indicates that clinical decision-making for early mobilization is influenced by neurological severity, cardio-respiratory status, staffing, and local stroke unit protocols, underscoring the importance of evidence-based algorithms that translate available evidence into reproducible bedside practice (6).

For older AIS patients, the rationale for early mobilization is strengthened by the intersecting risks of sarcopenia, frailty, aspiration, venous thromboembolism, and pressure injury during prolonged bed rest. Recent work has highlighted the high prevalence and clinical relevance of post-stroke frailty and its association with reduced rehabilitation adherence and poorer recovery, suggesting that older adults may particularly benefit from structured, monitored programs that balance early activation with safety thresholds (7, 8). Observational data also indicate age-related differences in rehabilitation responsiveness, reinforcing the need for age-conscious inpatient strategies that optimize ADL and motor gains without excessive early physiological stress (9). Recent literature has highlighted increasing interest in early mobilization after acute ischemic stroke; however, evidence remains limited regarding how a structured early mobilization pathway can be operationalized within routine stroke-unit practice for older patients (10, 11). Although current studies and guidelines support early rehabilitation, uncertainty persists about the optimal timing, dose, and safety governance of early out-of-bed activity, particularly in individuals with multimorbidity and greater clinical complexity (12). Furthermore, many previous investigations have focused on efficacy under controlled or heterogeneous intervention conditions, leaving insufficient evidence on whether a protocolized, safety-guided early mobilization algorithm integrated into standard care can be feasibly implemented and influence short-term functional recovery and in-hospital outcomes in this high-risk population (13, 14).

Despite emerging clinical reports suggesting that structured early rehabilitation models may improve neurological function, activities of daily living, and quality of life, evidence evaluating the integration of an evidence-based early mobilization pathway into routine conventional rehabilitation for older patients with acute ischemic stroke remains limited (15). To address this gap, the present study evaluated the implementation of an evidence-based early mobilization pathway added to conventional rehabilitation in older patients with acute ischemic stroke.

## 2 Methods

### 2.1 Study design

This retrospective cohort study enrolled consecutive older patients diagnosed with acute ischemic stroke at our hospital between January 2023 and January 2025. The study was designed and reported in accordance with the Strengthening the Reporting of Observational Studies in Epidemiology (STROBE) guidelines to ensure transparency and completeness of reporting (16). Eligible participants were aged ≥60 years (17, 18), met established diagnostic criteria for acute ischemic stroke with imaging confirmation (CT and/or MRI), were admitted within an appropriate acute time window after symptom onset, had stable vital signs to permit rehabilitation assessment, and had complete clinical and rehabilitation records. Patients were excluded if they had intracerebral or subarachnoid hemorrhage, transient ischemic attack without infarction, severe pre-stroke functional dependence (e.g., markedly elevated baseline mRS), coma or rapidly progressive neurological deterioration precluding mobilization, unstable cardiopulmonary conditions, active severe infection, recent major surgery or trauma, musculoskeletal disorders contraindicating early mobilization, or incomplete key outcome data. According to the actual rehabilitation regimen implemented during different institutional periods, patients receiving routine conventional rehabilitation from January 2023 to January 2024 were assigned to the control group, whereas those treated from January 2024 to January 2025 with an evidence-based early mobilization program in addition to conventional rehabilitation were assigned to the observation group. Informed consent was obtained from all participants. The study was approved by the hospital’s ethics committee and conducted in accordance with relevant guidelines and the Declaration of Helsinki. All data were extracted from the same institutional electronic medical record system, and both rehabilitation protocols were delivered by the same multidisciplinary stroke team using standardized clinical pathways throughout the study period. All data were anonymized prior to analysis to ensure participant confidentiality.

### 2.2 Rehabilitation protocols

Patients in the control group received routine conventional rehabilitation as implemented at our center between January 2023 and January 2024. The protocol was initiated after clinical stabilization of neurological status and vital signs, typically within 24–72 h of admission, and was delivered by a multidisciplinary stroke rehabilitation team. The conventional program included: (1) standardized positioning, limb alignment, and scheduled turning to prevent pressure injury, shoulder subluxation, and joint malalignment, with routine prophylaxis for immobility-related complications according to institutional practice; (2) passive and active-assisted range of motion for affected upper and lower limbs, generally 1–2 times daily with 10–15 repetitions per major joint, progressing to active ROM as tolerated to reduce contracture risk; (3) bed-based low-intensity therapeutic exercises such as ankle pumps, isometric quadriceps/gluteal activation, bridging, and gentle trunk control training, typically once daily for 20–30 min; (4) sitting balance training at the bedside and assisted transfers (e.g., bed-to-chair) initiated based on routine clinical assessment without a structured early mobilization algorithm; (5) assisted standing and short-distance ambulation with parallel bars or walking aids when patients achieved sufficient sitting and standing tolerance, combined with occupational therapy targeting basic activities of daily living; and (6) routine swallowing and communication rehabilitation for patients with dysphagia or aphasia when indicated. Overall, patients commonly received one structured therapist-led session daily (approximately 20–40 min), supplemented by nursing-assisted limb exercises and ADL guidance 5–6 days per week during hospitalization.

Patients in the observation group received the same conventional rehabilitation components between January 2024 and January 2025, with the addition of a structured evidence-based early mobilization (EBEM) protocol aimed at promoting earlier and safer out-of-bed activity. The EBEM framework comprised: (1) standardized eligibility screening for early mobilization based on stable clinical status, with initiation as soon as feasible, commonly within 24 h of admission after confirmation of neurological and hemodynamic stability by the attending team; (2) structured safety monitoring before, during, and after each session, with predefined stop criteria for new or worsening neurological deficits, clinically significant cardiopulmonary instability, symptomatic blood pressure abnormalities, chest pain, syncope, severe dizziness, or oxygen desaturation; (3) a stepwise mobilization pathway including graded bed-level activation, supported sitting at the edge of the bed, assisted bed-to-chair transfer, supported standing, and early gait training, with advancement contingent on tolerance and absence of adverse events; (4) higher-frequency, dose-controlled mobilization sessions of short duration initially (typically 10–20 min per session, 1–3 sessions/day), progressing in time and complexity according to patient response; and (5) integration of early mobilization with ADL-oriented task practice, emphasizing trunk control, upper-limb functional use, and caregiver-involved safe activity after standardized education. The observation protocol therefore differed primarily by the presence of an evidence-based algorithm that specified timing, safety thresholds, progression criteria, and higher-frequency out-of-bed activities, while maintaining core conventional rehabilitation content to support between-group comparability.

### 2.3 Data collection

Data were retrospectively extracted from the hospital electronic medical record system for patients admitted with acute ischemic stroke between January 2023 and January 2025. A standardized, prespecified case report form was used to ensure consistent variable definitions and data completeness. Two investigators independently performed data extraction and cross-checked entries, with discrepancies resolved through consensus and review of source documents. Demographic variables included age, sex, and body mass index. Stroke-related clinical data comprised time from symptom onset to admission, baseline neurological severity assessed by the National Institutes of Health Stroke Scale (NIHSS) at admission, and infarct territory determined from neuroimaging reports (anterior circulation, posterior circulation, or multiple territories). Vascular comorbidities and risk factors were obtained from documented medical history and admission diagnoses, including hypertension, diabetes mellitus, atrial fibrillation, coronary artery disease, and previous stroke. Process indicators of early mobilization were extracted from rehabilitation and nursing records, including time to first out-of-bed mobilization, proportions mobilized within 24 and 48 h, session frequency, and mobilization dose (duration per session and cumulative daily and in-hospital mobilization time). The highest mobilization stage achieved during hospitalization was recorded using a predefined 5-stage framework (bed-level activity, sitting at the edge of the bed, bed-to-chair transfer, standing, and ambulation). In the EBEM group, feasibility metrics were additionally collected, including delayed or withheld sessions and the primary reasons for non-delivery. Functional status was evaluated using standardized rehabilitation instruments recorded in routine clinical practice. Activities of daily living and motor and balance function were assessed at admission and discharge using the Barthel Index (BI), Fugl–Meyer Assessment motor score (FMA-motor), and Berg Balance Scale (BBS), and change scores were calculated. Functional prognosis at discharge was assessed using the modified Rankin Scale (mRS), with favorable outcome defined as mRS 0–2. Gait performance at discharge, when documented in routine rehabilitation assessment, included walking distance and walking speed measured under therapist supervision using institutional standard procedures. In-hospital process indicators included length of stay and ICU transfer. Thirty-day readmission was obtained from institutional records and follow-up documentation. Safety outcomes and complications were captured from physician and nursing documentation and discharge diagnoses, including pneumonia, lower-extremity deep vein thrombosis and/or pulmonary embolism, pressure injury, and urinary tract infection; time to pneumonia was recorded among affected patients. Patients lacking key baseline or outcome data were excluded according to predefined criteria. All in-hospital complications were identified from physician documentation, nursing records, and discharge diagnoses in the institutional electronic medical record system. Diagnostic and surveillance practices remained unchanged throughout the study period, and no new screening protocols were introduced between calendar periods. Pneumonia was diagnosed based on compatible clinical features with radiographic findings and/or antibiotic treatment. Venous thromboembolism (deep vein thrombosis and/or pulmonary embolism) was confirmed by objective imaging when clinically suspected. Pressure injury was diagnosed and staged according to routine nursing and physician assessment. Urinary tract infection was diagnosed based on clinical symptoms with supportive laboratory findings.

### 2.4 Statistical analysis

All statistical analyses were performed using IBM SPSS Statistics version 26.0 (IBM Corp., Armonk, NY, United States). Continuous variables are presented as mean ± standard deviation and were compared using independent-samples *t*-tests after assessment of distributional characteristics. Categorical variables are expressed as number (percentage) and were compared using the *χ*^2^ test or Fisher’s exact test, as appropriate. The primary outcome was favorable functional status at discharge, defined as a mRS score of 0–2. Between-group differences in favorable outcome rates were assessed using the *χ*^2^ test. Neurological improvement was evaluated by change in NIHSS scores from admission to discharge. Secondary outcomes, including the Barthel Index, Fugl–Meyer Assessment motor score, Berg Balance Scale, walking distance, walking speed, length of stay, and in-hospital complications, were compared between groups using appropriate parametric or categorical tests. To address potential confounding, multivariable logistic regression was performed to evaluate the independent association between evidence-based early mobilization and favorable outcome, adjusting for clinically relevant covariates. Results are reported as odds ratios with 95% confidence intervals. Prespecified subgroup analyses were conducted with interaction testing. Sensitivity analyses included exclusion of patients with potentially influential clinical profiles, alternative multivariable model specifications, and propensity score–based approaches (matching and inverse probability of treatment weighting). Additional analyses included ordinal logistic regression of the full mRS distribution and models incorporating length of stay as a covariate to assess the robustness of discharge-based outcomes. All tests were two-sided, with *p* < 0.05 considered statistically significant.

## 3 Results

### 3.1 Patient screening and group allocation

Between January 2023 and January 2025, consecutive older patients admitted to our hospital with a diagnosis of AIS were retrospectively screened. A total of 230 eligible admissions of older patients with AIS were assessed for inclusion. Of these, 53 patients were excluded according to the predefined eligibility criteria, primarily due to failure to meet the inclusion criteria (*n* = 22), contraindications to early mobilization or rehabilitation (*n* = 18), and incomplete clinical records or inability to complete outcome assessment (*n* = 13). Ultimately, 177 patients were included in the final analysis and were allocated based on the actual rehabilitation regimen implemented during two institutional periods: 86 patients who received routine conventional rehabilitation between January 2023 and January 2024 were assigned to the control group, and 91 patients who received an evidence-based early mobilization program in addition to conventional rehabilitation between January 2024 and January 2025 were assigned to the observation group.

### 3.2 Baseline demographic and clinical characteristics

The control group (*n* = 86) and observation group (*n* = 91) were compared in terms of demographic characteristics, stroke-related clinical indicators, comorbidities and vascular risk factors, infarct territory distribution, and baseline functional status at admission. No statistically significant between-group differences were observed in age, sex, or body mass index. Onset-to-admission time and baseline neurological severity assessed by the National Institutes of Health Stroke Scale were also comparable. The distribution of infarct territories did not differ between groups. In addition, the prevalence of major vascular comorbidities, including hypertension, diabetes mellitus, atrial fibrillation, coronary artery disease, and previous stroke, was balanced. Baseline functional measures on admission, including the Barthel Index, Fugl–Meyer Assessment motor score, and Berg Balance Scale, showed no significant differences (Table 1).

**Table 1:** Baseline demographic and clinical characteristics of older patients with AIS.

Variable	Control group (*n* = 86)	Observation group (*n* = 91)	Test statistic	*p*-value
Demographic variables				
Age, years	69.8 ± 6.4	70.3 ± 6.0	*t* = −0.54	0.593
Male sex, *n* (%)	49 (57.0)	53 (58.2)	*χ*^2^ = 0.03	0.865
BMI, kg/m^2^	24.2 ± 2.7	24.4 ± 3.3	*t* = −0.44	0.659
Stroke-related clinical indicators				
Onset-to-admission time, h	9.6 ± 4.1	9.9 ± 4.4	*t* = −0.47	0.639
Baseline NIHSS score	8.7 ± 3.0	8.2 ± 2.9	*t* = 1.13	0.262
Infarct territory, *n* (%)			*χ*^2^ = 0.08	0.961
Anterior circulation	54 (62.8)	59 (64.8)		
Posterior circulation	24 (27.9)	24 (26.4)		
Multiple territories	8 (9.3)	8 (8.8)		
Comorbidities and vascular risk factors				
Hypertension, *n* (%)	57 (66.3)	64 (70.3)	*χ*^2^ = 0.34	0.562
Diabetes mellitus, *n* (%)	25 (29.1)	30 (33.0)	*χ*^2^ = 0.31	0.576
Atrial fibrillation, *n* (%)	16 (18.6)	20 (22.0)	*χ*^2^ = 0.31	0.577
Coronary artery disease, *n* (%)	19 (22.1)	24 (26.4)	*χ*^2^ = 0.44	0.507
Previous stroke, *n* (%)	13 (15.1)	17 (18.7)	*χ*^2^ = 0.40	0.527
Baseline functional status on admission				
Baseline BI score	44.7 ± 11.5	45.8 ± 9.6	*t* = −0.69	0.492
Baseline FMA-motor score	41.5 ± 9.1	40.5 ± 9.5	*t* = 0.72	0.475
Baseline BBS score	18.8 ± 7.1	18.5 ± 6.7	*t* = 0.29	0.773

AIS, acute ischemic stroke; BMI, body mass index; NIHSS, National Institutes of Health Stroke Scale; BI, Barthel Index; FMA, Fugl–Meyer Assessment; BBS, Berg Balance Scale.

### 3.3 Implementation of early mobilization

Implementation metrics demonstrated clear differences between groups (Table 2). Patients in the EBEM group were mobilized substantially earlier, with a shorter time to first out-of-bed mobilization (19.8 ± 10.6 vs. 46.3 ± 18.7 h, *p* < 0.001), and a higher proportion mobilized within 24 h (70.3% vs. 30.2%) and 48 h (90.1% vs. 64.0%) (both *p* < 0.001). Mobilization frequency was higher in the EBEM group, with more sessions per day and a greater total number of sessions during hospitalization (both *p* < 0.001). Although the duration of individual sessions was shorter, the EBEM group achieved greater cumulative mobilization time per day and during hospitalization (both *p* ≤ 0.001). Consistent with these findings, patients receiving EBEM reached higher mobilization stages during hospitalization (Supplementary Table S1), and ambulation training was achieved more frequently. In the EBEM group, early mobilization was feasible in most patients, with 70.3% mobilized within 24 h and 90.1% within 48 h; delayed or withheld sessions occurred in 30.8% of patients, most commonly due to hemodynamic instability or scheduled medical procedures (Supplementary Table S2).

**Table 2:** Implementation of early mobilization during hospitalization (timing, frequency, and dose).

Variable	Control group (*n* = 86)	EBEM group (*n* = 91)	Test statistic	*p*-value
Timing				
First out-of-bed mobilization, hours from admission	46.3 ± 18.7	19.8 ± 10.6	*t* = 11.14	<0.001
Mobilized within 24 h, *n* (%)	26 (30.2)	64 (70.3)	*χ*^2^ = 28.0	<0.001
Mobilized within 48 h, *n* (%)	55 (64.0)	82 (90.1)	*χ*^2^ = 16.8	<0.001
Frequency				
Mobilization sessions per day, mean ± SD	0.9 ± 0.4	2.1 ± 0.7	*t* = −13.3	<0.001
Total mobilization sessions during hospitalization, mean ± SD	10.8 ± 5.2	22.4 ± 8.6	*t* = −10.6	<0.001
Dose (duration/intensity)				
Duration per mobilization session, min	25.1 ± 8.0	15.6 ± 5.4	*t* = 9.1	<0.001
Total mobilization time per day, min	22.1 ± 10.2	32.8 ± 12.4	*t* = −6.0	<0.001
Total mobilization time during hospitalization, min	282 ± 148	364 ± 172	*t* = −3.3	0.001

Values are presented as mean ± SD or *n* (%). Out-of-bed mobilization refers to the first documented session involving sitting at the edge of bed, bed-to-chair transfer, standing, or ambulation under supervision. EBEM, evidence-based early mobilization.

### 3.4 Functional prognostic outcomes and neurological improvement

At discharge, functional status was assessed using the mRS. The overall distribution of mRS scores did not differ significantly between groups (*χ*^2^ = 8.76, *p* = 0.188); however, the proportion of patients achieving a favorable functional outcome (mRS 0–2) was higher in the observation group than in the control group (54.9% vs. 34.9%; *χ*^2^ = 6.40, *p* = 0.011). Neurological recovery was further evaluated by changes in NIHSS from admission to discharge. Baseline NIHSS scores were comparable between the two groups (8.63 ± 3.23 vs. 8.43 ± 2.87; *p* = 0.676), while discharge NIHSS was lower in the observation group (3.95 ± 2.81 vs. 5.05 ± 3.21; *p* = 0.016). The observation group demonstrated a greater reduction in NIHSS during hospitalization (4.66 ± 1.27 vs. 3.67 ± 1.31; *t* = −5.06, *p* < 0.001) (Table 3). In multivariable logistic regression adjusting for age, sex, baseline NIHSS, and major vascular comorbidities, evidence-based early mobilization remained independently associated with favorable functional outcome (adjusted OR = 3.57, 95% CI 1.61–7.93, *p* = 0.002), whereas older age and higher baseline NIHSS were associated with lower odds of achieving mRS 0–2 at discharge (Table 4).

**Table 3:** Functional outcomes and neurological improvement at discharge.

Outcome	Control group (*n* = 86)	Observation group (*n* = 91)	Test statistic	*p*-value
mRS at discharge, *n* (%)			*χ*^2^ = 8.76	0.188
0	4 (4.7)	10 (11.0)		
1	12 (14.0)	21 (23.1)		
2	14 (16.3)	19 (20.9)		
3	18 (20.9)	13 (14.3)		
4	19 (22.1)	17 (18.7)		
5	13 (15.1)	8 (8.8)		
6	6 (7.0)	3 (3.3)		
Favorable functional outcome (mRS 0–2), *n* (%)	30 (34.9)	50 (54.9)	*χ*^2^ = 6.40	0.011
NIHSS score				
Baseline NIHSS, mean ± SD	8.63 ± 3.23	8.43 ± 2.87	*t* = 0.42	0.676
Discharge NIHSS, mean ± SD	5.05 ± 3.21	3.95 ± 2.81	*t* = 2.44	0.016
NIHSS change (baseline → discharge), mean ± SD	3.67 ± 1.31	4.66 ± 1.27	*t* = −5.06	<0.001

mRS, modified Rankin Scale; NIHSS, National Institutes of Health Stroke Scale; SD, standard deviation.

**Table 4:** Multivariable logistic regression for favorable functional outcome (mRS 0–2) at discharge.

Variable	Adjusted OR	95% CI	*p*-value
Group (observation vs. control)	3.57	1.61–7.93	0.002
Age (per 1-year increase)	0.86	0.81–0.92	<0.001
Male sex	1.36	0.64–2.90	0.430
Baseline NIHSS (per 1-point increase)	0.64	0.55–0.76	<0.001
Hypertension	0.87	0.39–1.95	0.730
Diabetes mellitus	0.74	0.32–1.68	0.469
Atrial fibrillation	1.32	0.55–3.21	0.536
Coronary artery disease	1.19	0.52–2.73	0.680
Previous stroke	0.68	0.24–1.98	0.483

OR, odds ratio; CI, confidence interval; NIHSS, National Institutes of Health Stroke Scale.

### 3.5 Secondary outcomes: ADL/motor function, balance/gait performance, and in-hospital course

Patients receiving evidence-based early mobilization demonstrated better secondary functional outcomes at discharge. Compared with the control group, the observation group achieved higher BI and FMA-motor scores at discharge and exhibited larger improvements from baseline, indicating greater gains in ADL independence and motor recovery (all *p* < 0.001). Balance and gait performance also favored the observation group, with higher BBS at discharge and greater BBS improvement, accompanied by longer walking distance and faster walking speed (all *p* < 0.001). Regarding in-hospital course, length of stay was shorter in the observation group (*p* = 0.010), and ICU transfer occurred less frequently (*p* = 0.045), while 30-day readmission rates were comparable between groups (*p* = 0.484) (Table 5).

**Table 5:** Secondary outcomes at discharge and during hospitalization.

Outcome	Control group (*n* = 86)	Observation group (*n* = 91)	Test statistic	*p*-value
ADL and motor function				
BI at discharge	72.1 ± 12.4	79.0 ± 11.6	*t* = −3.82	<0.001
BI change (baseline → discharge)	27.4 ± 10.2	33.2 ± 9.8	*t* = −3.85	<0.001
FMA-motor at discharge	57.8 ± 9.5	63.1 ± 9.2	*t* = −3.77	<0.001
FMA-motor change (baseline → discharge)	16.3 ± 7.0	22.6 ± 7.2	*t* = −5.90	<0.001
Balance and gait performance				
BBS at discharge	34.2 ± 8.0	38.9 ± 7.6	*t* = −4.00	<0.001
BBS change (baseline → discharge)	15.4 ± 6.1	20.4 ± 6.0	*t* = −5.49	<0.001
Walking distance at discharge, m	128 ± 52	156 ± 55	*t* = −3.48	<0.001
Walking speed at discharge, m/s	0.62 ± 0.18	0.72 ± 0.19	*t* = −3.60	<0.001
In-hospital process indicators				
Length of stay, days	12.8 ± 4.6	11.1 ± 4.1	*t* = 2.59	0.010
ICU transfer during hospitalization, *n* (%)	11 (12.8)	4 (4.4)	*χ*^2^ = 4.02	0.045
30-day readmission, *n* (%)	7 (8.1)	5 (5.5)	*χ*^2^ = 0.49	0.484

ADL, activities of daily living; BI, Barthel Index; FMA, Fugl–Meyer Assessment; BBS, Berg Balance Scale; ICU, intensive care unit.

### 3.6 Complications and clinical safety outcomes

Pneumonia occurred in 18 patients (20.9%) in the control group and 9 patients (9.9%) in the observation group, indicating a lower incidence in patients receiving evidence-based early mobilization (*χ*^2^ = 4.17, *p* = 0.041). Among patients who developed pneumonia, the time from admission to diagnosis was comparable between groups (4.2 ± 1.8 vs. 4.5 ± 1.6 days; *t* = −0.44, *p* = 0.665). The incidence of lower-extremity venous thromboembolism, defined as DVT and/or PE, was also lower in the observation group than in the control group (2.2% vs. 10.5%; *χ*^2^ = 5.18, *p* = 0.023). Similarly, pressure injury was less frequent in the observation group (1.1% vs. 7.0%; *χ*^2^ = 4.02, *p* = 0.045). In contrast, urinary tract infection rates did not differ significantly between groups (8.8% vs. 11.6%; *χ*^2^ = 0.39, *p* = 0.533) (Table 6).

**Table 6:** In-hospital complications and safety outcomes.

Complication	Control group (*n* = 86)	Observation group (*n* = 91)	Test statistic	*p*-value
Pneumonia, *n* (%)	18 (20.9)	9 (9.9)	*χ*^2^ = 4.17	0.041
Time to pneumonia, days	4.2 ± 1.8	4.5 ± 1.6	*t* = −0.44	0.665
Lower-extremity DVT/PE, *n* (%)	9 (10.5)	2 (2.2)	*χ*^2^ = 5.18	0.023
Pressure injury, *n* (%)	6 (7.0)	1 (1.1)	*χ*^2^ = 4.02	0.045
Urinary tract infection, *n* (%)	10 (11.6)	8 (8.8)	*χ*^2^ = 0.39	0.533

DVT, deep vein thrombosis; PE, pulmonary embolism.

### 3.7 Subgroup analyses

Across prespecified subgroups defined by baseline stroke severity, age, atrial fibrillation, diabetes mellitus, and multimorbidity burden, the association between evidence-based early mobilization and favorable functional outcome (mRS 0–2) was generally directionally consistent. However, formal tests for interaction were not significant for any subgroup factor (all *P* for interaction > 0.05), providing no evidence that the treatment effect differed across these strata (Table 7).

**Table 7:** Subgroup analyses of favorable functional outcome (mRS 0–2) at discharge.

Factor	Subgroup	Control favorable *n*/*N* (%)	Observation favorable *n*/*N* (%)	OR (95% CI)	*χ* ^2^	*p*-value	*P* for interaction
Stroke severity (baseline NIHSS)	NIHSS ≤8	24/50 (48.0)	36/55 (65.5)	2.05 (0.94–4.50)	3.26	0.071	0.526
	NIHSS ≥9	6/36 (16.7)	14/36 (38.9)	3.18 (1.06–9.59)	4.43	0.035	
Age	60–74 years	23/58 (39.7)	36/60 (60.0)	2.28 (1.09–4.77)	4.88	0.027	0.907
	≥75 years	7/28 (25.0)	14/31 (45.2)	2.47 (0.81–7.50)	2.61	0.106	
Atrial fibrillation	Absent	27/70 (38.6)	43/71 (60.6)	2.45 (1.24–4.81)	6.82	0.009	0.957
	Present	3/16 (18.8)	7/20 (35.0)	2.33 (0.49–11.06)	1.17	0.279	
Diabetes mellitus	Absent	23/61 (37.7)	35/61 (57.4)	2.22 (1.08–4.59)	4.73	0.030	0.832
	Present	7/25 (28.0)	15/30 (50.0)	2.57 (0.83–7.95)	2.75	0.097	
Multimorbidity (≥2 comorbidities)	No	20/46 (43.5)	32/49 (65.3)	2.45 (1.07–5.60)	4.56	0.033	0.896
	Yes	10/40 (25.0)	18/42 (42.9)	2.25 (0.88–5.77)	2.91	0.088	

mRS, modified Rankin Scale; NIHSS, National Institutes of Health Stroke Scale; OR, odds ratio; CI, confidence interval.

### 3.8 Sensitivity analyses

Across multiple sensitivity analyses, the association between evidence-based early mobilization and favorable functional outcome at discharge remained stable. After excluding patients with potentially strong confounding profiles such as extremely severe baseline status or very short hospitalization, the observation group continued to demonstrate a higher rate of mRS 0–2 compared with the control group (54.7% vs. 35.0%; OR = 2.24, 95% CI 1.20–4.18; *p* = 0.017). The effect size was consistent when alternative multivariable specifications were applied, with the group effect remaining significant in both the core-adjusted model and the fully adjusted model. Propensity score approaches yielded concordant results: in the matched cohort, the observation group maintained a higher proportion of favorable outcomes (55.7% vs. 34.3%; OR = 2.41, 95% CI 1.22–4.77; *p* = 0.017), and the IPTW-weighted analysis produced a comparable adjusted estimate (Table 8). Propensity score model specification and balance diagnostics are provided in Supplementary Appendix S3 and Supplementary Tables S4, S5.

**Table 8:** Sensitivity analyses for favorable functional outcome (mRS 0–2) at discharge.

Analysis strategy	Control group	Observation group	Effect estimate	Test statistic	*p*-value
Primary analysis (unadjusted)	30/86 (34.9)	50/91 (54.9)	OR = 2.28 (95% CI 1.24–4.17)	*χ*^2^ = 6.40	0.011
Excluding potential key confounders*	28/80 (35.0)	47/86 (54.7)	OR = 2.24 (95% CI 1.20–4.18)	*χ*^2^ = 5.69	0.017
Core-adjusted model^†^	—	—	aOR = 3.10 (95% CI 1.50–6.39)	Wald	0.002
Fully adjusted model^‡^	—	—	aOR = 3.57 (95% CI 1.61–7.93)	Wald	0.002
Propensity score matching (1:1)	24/70 (34.3)	39/70 (55.7)	OR = 2.41 (95% CI 1.22–4.77)	*χ*^2^ = 5.66	0.017
IPTW-weighted analysis	—	—	aOR = 2.95 (95% CI 1.45–6.02)	Wald	0.003

mRS, modified Rankin Scale; OR, odds ratio; aOR, adjusted odds ratio; CI, confidence interval; IPTW, inverse probability of treatment weighting.

*Exclusion criteria example: patients with extremely severe stroke at baseline and/or very short hospitalization (e.g., length of stay <3 days), leaving 80 patients in the control group and 86 in the observation group for this analysis.

^†^Core-adjusted model included group, age, sex, and baseline NIHSS.

^‡^Fully adjusted model additionally included major vascular comorbidities (e.g., hypertension, diabetes mellitus, atrial fibrillation, coronary artery disease, and previous stroke).

### 3.9 Sensitivity analyses accounting for discharge timing and ordinal mRS distribution

To further examine the robustness of the primary outcome and reconcile differences between analytical approaches, additional analyses were conducted. Although the overall distribution of mRS scores did not differ significantly between groups using the chi-square test, an ordinal logistic regression shift analysis demonstrated that evidence-based early mobilization was significantly associated with a favorable shift across the full mRS spectrum (common odds ratio 2.12, 95% confidence interval 1.32–3.41; *p* = 0.002). The apparent discrepancy arises because the chi-square test assesses categorical distribution differences without accounting for ordinal structure, whereas ordinal logistic regression leverages the ordered nature of mRS scores and may have greater sensitivity to detect proportional shifts across disability levels. Furthermore, when length of stay was incorporated as an additional covariate in the multivariable model, evidence-based early mobilization remained independently associated with favorable functional outcome (adjusted odds ratio 3.21, 95% confidence interval 1.44–7.15; *p* = 0.004), suggesting that the primary findings were unlikely to be explained solely by differences in discharge timing. These results support the robustness of the main analysis despite the discharge-based assessment of mRS (Supplementary Table S6).

## 4 Discussion

In-hospital mobilization is a core component of contemporary stroke-unit rehabilitation, yet the optimal timing and delivered dose of early out-of-bed activity in older adults remain variably implemented in routine practice, and empirical evidence linking structured early mobilization pathways to short-term functional outcomes is still limited (19, 20). In this retrospective, period-based cohort of older patients with acute ischemic stroke managed within the same institution, implementation of an evidence-based early mobilization program in addition to conventional rehabilitation was associated with a higher likelihood of favorable functional outcome at discharge (mRS 0–2), alongside greater neurological improvement (larger NIHSS reduction) and better discharge performance in ADL independence, motor recovery, balance, and gait. The EBEM cohort also experienced a shorter length of stay and lower ICU transfer rates, and showed fewer immobility-related complications (pneumonia, venous thromboembolism, and pressure injury) without an apparent increase in urinary tract infection. The novelty of this study lies in evaluating a standardized, algorithm-driven EBEM pathway under routine stroke-ward conditions and, importantly, quantifying the delivered implementation metrics (timing, frequency, cumulative dose, stage progression, and feasibility barriers), while also supporting robustness through complementary adjustment and sensitivity analyses. Clinically, these findings suggest that operationalizing an evidence-based early mobilization pathway may provide incremental benefit beyond usual rehabilitation by accelerating functional recovery and reducing immobility-related harms during hospitalization, offering a pragmatic framework that can be embedded into standardized stroke-unit workflows for older patients.

In this retrospective cohort of older adults with acute ischemic stroke, implementation of an EBEM program in addition to conventional rehabilitation was associated with a higher likelihood of achieving a favorable functional outcome at discharge. Although the overall discharge mRS distribution did not differ significantly between groups using a Pearson *χ*^2^ test, ordinal logistic regression demonstrated a significant favorable shift across the full mRS spectrum (common OR 2.12, 95% CI 1.32–3.41). The EBEM cohort also had a higher proportion of patients achieving mRS 0–2 at discharge (54.9% vs. 34.9%), which persisted after multivariable adjustment (adjusted OR 3.57, 95% CI 1.61–7.93). Concordant improvements across secondary endpoints further support clinical plausibility: EBEM patients showed greater neurological improvement (larger NIHSS reduction), better activities of daily living independence (Barthel Index), improved motor recovery (Fugl–Meyer motor score), better balance (Berg Balance Scale), and superior gait performance (greater walking distance and faster speed) at discharge. EBEM also resulted in meaningful differences in care delivery, including earlier out-of-bed mobilization (~20 vs. ~46 h), higher session frequency, and greater cumulative mobilization time, despite shorter per-session duration. A higher maximum mobilization stage and more frequent ambulation training indicate facilitated progression along a structured mobility continuum. Feasibility was acceptable in routine practice, with most patients mobilized within 48 h and delays occurring in a minority, primarily due to hemodynamic instability or competing procedures, consistent with safety considerations in acute stroke care. Additionally, EBEM was associated with lower rates of in-hospital complications such as pneumonia, venous thromboembolism, and pressure injury, without an increase in other adverse events. While causality cannot be inferred from this observational design, the consistency across implementation metrics, functional outcomes, and immobility-related complications suggests that EBEM may contribute to clinically meaningful benefits during the acute hospitalization period (21, 22).

The robustness of the primary finding was supported by a structured set of subgroup and sensitivity analyses, while also underscoring important interpretive constraints. Across prespecified subgroups (baseline stroke severity, age strata, atrial fibrillation, diabetes mellitus, and multimorbidity), effect estimates were directionally consistent, and formal interaction tests were uniformly non-significant, providing no statistical evidence that the association differed across strata; accordingly, subgroup results should be interpreted as exploratory and hypothesis-generating. Sensitivity analyses further addressed residual confounding and model dependence. Excluding potentially influential clinical profiles (e.g., extremely severe baseline status or very short hospitalization) did not materially change the association with favorable outcome. Alternative covariate specifications yielded consistent adjusted effects, and propensity score approaches (1:1 matching and stabilized inverse probability of treatment weighting) produced concordant estimates with adequate post-adjustment covariate balance, supporting internal consistency across analytic frameworks. Given concerns that discharge mRS is influenced by discharge timing and practices, particularly because length of stay differed between groups, additional analyses were appropriately used to mitigate this bias. A shift analysis using ordinal logistic regression across the full mRS range (0–6) demonstrated a significant shift toward better outcomes, suggesting that the association was not confined to a dichotomized endpoint. Moreover, inclusion of length of stay as an additional covariate did not attenuate the EBEM–outcome association, and length of stay itself was not independently associated with favorable outcome in the fully adjusted model, collectively arguing against discharge timing as the sole explanation for the observed effect. Nevertheless, residual confounding remains possible, especially because exposure assignment was based on calendar periods.

A plausible explanation for the observed functional benefits relates to the interaction between early, graded physical activity and activity-dependent neuroplasticity during the acute and subacute phases of stroke recovery (23, 24). Contemporary rehabilitation frameworks emphasize that appropriately dosed early task exposure may harness heightened neural responsiveness while mitigating immobility-related complications such as deconditioning, sarcopenia, orthostatic intolerance, and delirium. Recent evidence has moved beyond the simplistic “early versus late” paradigm toward optimizing timing, intensity, frequency, and patient selection (10, 23). Systematic reviews suggest that initiation after the first 24 h in clinically stable patients, delivered as short and repeated sessions one to three times daily, is most consistent with safety and functional benefit, and emerging meta-analyses indicate that individualized, closely monitored early rehabilitation within 48 h can improve neurological recovery and self-care outcomes while cautioning against excessive early intensity (3). These observations align with the present findings and support the concept that a structured, safety-guided and dose-controlled mobilization pathway, rather than indiscriminate high-intensity activity, may enhance early functional recovery and reduce immobility-related complications during hospitalization (25). From an implementation perspective, the study offers practical insights into safe delivery of early mobilization in routine care. The EBEM protocol incorporated standardized eligibility screening, predefined safety criteria, graded progression from bed-level activity to ambulation, and higher-frequency but shorter mobilization sessions. The high proportion of patients mobilized within 48 h and the clinically appropriate reasons for delayed sessions demonstrate the feasibility of protocolized early mobilization while maintaining individualized decision-making and physiologic monitoring. These findings support the adoption of structured early mobilization algorithms emphasizing early out-of-bed activity, dose titration, and interdisciplinary coordination. At the health-system level, EBEM represents a modifiable component of inpatient stroke management that may contribute to quality improvement and resource optimization (26). Prospective multicenter studies are needed to confirm generalizability, refine patient selection, and determine the optimal timing and dose of early mobilization across diverse clinical settings (13, 27).

However, while these findings provide important insights into the potential benefits of early mobilization, further analyses were conducted to explore the robustness of the primary outcome and account for potential confounding factors, particularly those related to discharge timing and length of stay. Although the overall distribution of mRS categories did not differ significantly between groups using a Pearson *χ*^2^ test, the ordinal logistic regression demonstrated a significant favorable shift across the full mRS spectrum. This apparent discrepancy likely reflects methodological differences between analytical approaches. The *χ*^2^ test evaluates categorical distribution differences without accounting for the ordered structure of the mRS, whereas ordinal regression leverages the ordinal nature of the scale and may provide greater statistical efficiency to detect proportional shifts across disability levels. Therefore, the shift analysis may be more sensitive to distributed improvements across multiple adjacent mRS categories rather than large changes concentrated within a single category threshold. In addition, a sensitivity analysis adjusting for length of stay was performed to account for potential confounding by differences in hospitalization duration. The association between evidence-based early mobilization and favorable functional outcome remained statistically significant after incorporating length of stay as a covariate, suggesting that the primary findings were not fully explained by discharge timing. However, given that length of stay may be influenced by the intervention itself and could lie on the causal pathway between early mobilization and discharge mRS, adjustment for length of stay may induce post-treatment bias. We therefore present the length of stay-adjusted model as a sensitivity analysis only and interpret these results cautiously. The persistence of the association suggests that the main findings are unlikely to be entirely driven by discharge timing, but causal interpretation remains inappropriate. Future studies may need to further refine this aspect to eliminate any potential bias.

This study has several strengths. It evaluated a pragmatic evidence-based early mobilization program embedded within routine stroke-unit care and assessed a comprehensive set of clinically meaningful outcomes, including functional status, neurological recovery, rehabilitation measures, gait performance, hospital course, and safety events. Baseline comparability across major demographic and clinical variables was confirmed, and the robustness of the primary findings was supported by prespecified subgroup analyses, multiple sensitivity analyses, and propensity score approaches. Several limitations should be acknowledged. The retrospective study introduces risks of selection bias and residual confounding. In addition, the period-based implementation design may be susceptible to calendar-time bias. Although no major changes in institutional stroke pathways, staffing, or rehabilitation resources occurred during the study period, temporal changes in broader stroke management cannot be fully excluded. The primary endpoint was discharge functional status, which may be influenced by discharge timing and institutional practices despite additional analyses adjusting for length of stay and evaluating the full modified Rankin Scale distribution. Outcome ascertainment relied on routinely collected clinical records and may therefore be subject to residual misclassification or documentation bias. Some secondary measures, including gait performance, were not available for all patients and may have introduced measurement bias. The absence of standardized long-term follow-up limits inference regarding durability of benefit beyond hospitalization. In addition, subgroup analyses were not powered to detect interaction effects and should be interpreted as exploratory and hypothesis generating. Finally, detailed dose–response relationships for mobilization exposure were not fully quantified across all participants, which limits mechanistic interpretation. Future prospective multicenter studies with standardized follow-up at fixed time points are needed to confirm generalizability, clarify causal effects, refine patient selection, and determine optimal timing, intensity, and dose of early mobilization. Further work should evaluate long-term functional recovery, quality of life, and cost effectiveness to inform broader implementation of structured early mobilization pathways in stroke-unit care.

## 5 Conclusion

This retrospective cohort study suggests that adding an evidence-based early mobilization program to conventional rehabilitation could be associated with improved short-term outcomes in older patients with acute ischemic stroke. The intervention was associated with higher rates of favorable functional status, greater neurological recovery, better ADL, motor, balance, and gait performance, shorter hospitalization, and fewer immobility-related complications. These findings support the potential role of structured, safety-guided early mobilization in acute stroke care.

## Data Availability

The raw data supporting the conclusions of this article will be made available by the authors, without undue reservation.

## References

[1] Chae J Celnik PA. Stroke rehabilitation. Phys Med Rehabil Clin N Am. (2015) 26:15–6. doi: 10.1016/j.pmr.2015.08.013, 26522913

[2] Jolliffe L Christie LJ Fearn N Nohrenberg M Liu R Williams JF . A systematic review of discrete choice experiments in stroke rehabilitation. Top Stroke Rehabil. (2024) 31:632–43. doi: 10.1080/10749357.2024.2312641, 38372124

[3] Lou Y Liu Z Ji Y Cheng J Zhao C Li L. Efficacy and safety of very early rehabilitation for acute ischemic stroke: a systematic review and meta-analysis. Front Neurol. (2024) 15:1423517. doi: 10.3389/fneur.2024.1423517, 39502386 PMC11534803

[4] Yen HC Pan GS Jeng JS Chen WS. Impact of early mobilization on patients with acute ischemic stroke treated with thrombolysis or thrombectomy: a randomized controlled trial. Neurorehabil Neural Repair. (2024):15459683241236443. doi: 10.1177/15459683241236443 38426480

[5] Li X Kong Y. Safety and efficacy of very early mobilization in acute stroke: a systematic review and meta-analysis. Arch Phys Med Rehabil. (2026) 107:333–52. doi: 10.1016/j.apmr.2025.10.021, 41242679

[6] Rethnam V Hayward KS Johns H Carvalho LB Churilov L Bernhardt J. Clinical and systems of care factors contributing to individual patient decision-making for early mobilization post-stroke. Front Stroke. (2023) 2:1293942. doi: 10.3389/fstro.2023.1293942, 41541077 PMC12802755

[7] Fan X Xia Y Xu S Jia S. A narrative review of interventions for post-stroke frailty: current advances and future directions. Front Neurol. (2025) 16:1592797. doi: 10.3389/fneur.2025.1592797, 40697579 PMC12279486

[8] Long Q Wu L Li Y Wu Y. Impact of frailty on rehabilitation exercise adherence in patients with ischemic stroke. Front Med. (2025) 12:1679267. doi: 10.3389/fmed.2025.1679267, 41281997 PMC12634566

[9] Tani T Kazuya W Onuma R Fushimi K Imai S. Age-related differences in the effectiveness of rehabilitation to improve activities of daily living in patients with stroke: a cross-sectional study. Ann Geriatr Med Res. (2024) 28:257–65. doi: 10.4235/agmr.24.0025, 38803998 PMC11467512

[10] Zhang M Wang Q Jiang Y Shi H Peng T Wang M. Optimization of early mobilization program for patients with acute ischemic stroke: an orthogonal design. Front Neurol. (2021) 12:645811. doi: 10.3389/fneur.2021.645811, 33912126 PMC8072335

[11] Singam A. Mobilizing Progress: a comprehensive review of the efficacy of early mobilization therapy in the intensive care unit. Cureus. (2024) 16:e57595. doi: 10.7759/cureus.57595, 38707138 PMC11069628

[12] Sepúlveda P Gallardo A Arriagada R González E Rocco PRM Battaglini D. Protocolized strategies to encourage early mobilization of critical care patients: challenges and success. Crit Care Sci. (2025) 37:e20250128. doi: 10.62675/2965-2774.20250128, 39936773 PMC11805459

[13] Filipska-Blejder K Jaracz K Ślusarz R. Efficacy and safety of early mobilization and factors associated with rehabilitation after stroke-review. J Clin Med. (2025) 14. doi: 10.3390/jcm14051585, 40095508 PMC11900172

[14] Maffassanti-Reyes M González-Sierra M Javier-Ormazábal A. Efficacy of early mobilization in stroke patients in relation to quality of life and level of dependency: a systematic review. Healthcare. (2025) 14. doi: 10.3390/healthcare14010078, 41517009 PMC12785253

[15] Wang H Ai Y Liang Y Zhu L Shi H Hou R. Efficacy of early rehabilitation nursing on the prognosis of China stroke patients: a systematic review and meta-analysis of randomized controlled trials. Geriatr Nurs. (2025) 64:103332. doi: 10.1016/j.gerinurse.2025.04.005, 40382238

[16] von Elm E Altman DG Egger M Pocock SJ Gøtzsche PC Vandenbroucke JP. The strengthening the reporting of observational studies in epidemiology (STROBE) statement: guidelines for reporting observational studies. J Clin Epidemiol. (2008) 61:344–9. doi: 10.1016/j.jclinepi.2007.11.008 18313558

[17] Noto S. Perspectives on aging and quality of life. Healthcare. (2023) 11. doi: 10.3390/healthcare11152131, 37570372 PMC10418952

[18] Abdullah JM Ismail A Yusoff MSB. Healthy ageing in Malaysia by 2030: needs, challenges and future directions. Malays J Med Sci. (2024) 31:1–13. doi: 10.21315/mjms2024.31.4.1, 39247109 PMC11376998

[19] Marzolini S Robertson AD Oh P Goodman JM Corbett D Du X . Aerobic training and mobilization early post-stroke: cautions and considerations. Front Neurol. (2019) 10:1187. doi: 10.3389/fneur.2019.01187, 31803129 PMC6872678

[20] Bernhardt J. Very early mobilization following acute stroke: controversies, the unknowns, and a way forward. Ann Indian Acad Neurol. (2008) 11:S88–s98. 35721448 PMC9204116

[21] So C Lage DE Slocum CS Zafonte RD Schneider JC. Utility of functional metrics assessed during acute care on hospital outcomes: a systematic review. PMR. (2019) 11:522–32. doi: 10.1002/pmrj.12013, 30758920 PMC10108704

[22] Olarte Parra C Bertizzolo L Schroter S Dechartres A Goetghebeur E. Consistency of causal claims in observational studies: a review of papers published in a general medical journal. BMJ Open. (2021) 11:e043339. doi: 10.1136/bmjopen-2020-043339, 34016660 PMC8141434

[23] de Mariana Aquino Miranda J Mendes Borges V Bazan R José Luvizutto G Sabrysna Morais Shinosaki J. Early mobilization in acute stroke phase: a systematic review. Top Stroke Rehabil. (2023) 30:157–68. doi: 10.1080/10749357.2021.2008595, 34927568

[24] Li X He Y Wang D Rezaei MJ. Stroke rehabilitation: from diagnosis to therapy. Front Neurol. (2024) 15:1402729. doi: 10.3389/fneur.2024.1402729, 39193145 PMC11347453

[25] Wei X Sun S Zhang M Zhao Z. A systematic review and meta-analysis of clinical efficacy of early and late rehabilitation interventions for ischemic stroke. BMC Neurol. (2024) 24:91. doi: 10.1186/s12883-024-03565-8, 38459477 PMC10921695

[26] Eapen BC Tran J Ballard-Hernandez J Buelt A Hoppes CW Matthews C . Stroke rehabilitation: synopsis of the 2024 U.S. department of veterans affairs and U.S. department of defense clinical practice guidelines. Ann Intern Med. (2025) 178:249–68. doi: 10.7326/ANNALS-24-02205, 39832369

[27] Viswambharan J Thahira SM Joseph S Maart S Morris LD Ferguson GD. Exploring the implementation of early mobilization of stroke patients in Qatar. Cureus. (2025) 17:e92798. doi: 10.7759/cureus.92798, 41122571 PMC12536945

