# Supplementary material for: Effects of evidence-based early mobilization on prognostic outcomes in older patients with acute ischemic stroke
Source: Front Public Health. 2026 Mar 16;14:1774045. doi: 10.3389/fpubh.2026.1774045 (PMC13033566; doi:10.3389/fpubh.2026.1774045)
Supplement: Supplementary file 1 [file Data_Sheet_1.docx]

Supplementary Table S1. Highest mobilization stage achieved during hospitalization

| Highest achieved stage | Control group (n = 86), n (%) | EBEM group (n = 91), n (%) |
| --- | --- | --- |
| Stage 1: Bed-level activity only | 10 (11.6) | 4 (4.4) |
| Stage 2: Sitting at the edge of the bed | 20 (23.3) | 10 (11.0) |
| Stage 3: Bed-to-chair transfer | 18 (20.9) | 15 (16.5) |
| Stage 4: Standing training | 22 (25.6) | 23 (25.3) |
| Stage 5: Ambulation training | 16 (18.6) | 39 (42.9) |
| **Overall comparison (Pearson χ² test)** | **χ² = 21.4 (df = 4)** | **p < 0.001** |

Notes: Highest achieved stage was defined as the maximum mobilization level documented at any time during hospitalization in rehabilitation and/or nursing records. The Pearson χ² test compares the overall distribution of mobilization stages between groups.

Staging: Stage 1, bed-level activity; Stage 2, sitting at the edge of the bed; Stage 3, bed-to-chair transfer; Stage 4, standing training; Stage 5, ambulation training.

Abbreviations: EBEM, evidence-based early mobilization.

Supplementary Table S2. Feasibility of EBEM implementation and primary reasons for delaying/withholding sessions (EBEM group only)

| Variable | EBEM group (n = 91) |
| --- | --- |
| Mobilized within 24 hours, n (%) | 64 (70.3) |
| Mobilized within 48 hours, n (%) | 82 (90.1) |
| Any delayed/withheld EBEM session during hospitalization, n (%) | 28 (30.8) |
| **Primary reason for delay/withholding (among those with ≥1 delayed/withheld session, n = 28), n (%)** |  |
| Hemodynamic instability (e.g., labile blood pressure, tachyarrhythmia) | 9 (32.1) |
| Neurological deterioration/new symptoms | 4 (14.3) |
| Medical procedures/tests (e.g., imaging, interventions) | 6 (21.4) |
| ICU transfer/need for higher-level monitoring | 3 (10.7) |
| Severe fatigue/poor tolerance (e.g., dizziness, nausea) | 4 (14.3) |
| Patient refusal/low cooperation | 2 (7.1) |

Notes: “Delayed/withheld” indicates that a planned EBEM mobilization session was postponed or not delivered due to documented clinical or operational reasons. Percentages in the first three rows are calculated using the full EBEM cohort (n = 91). Reasons for delay/withholding are summarized using the subgroup with ≥1 delayed/withheld session (n = 28); “primary reason” refers to the main documented reason per patient.

Abbreviations: EBEM, evidence-based early mobilization; ICU, intensive care unit.

**Supplementary Appendix S3. Propensity score analyses and diagnostics.**

**Propensity score analysis**

Propensity score (PS) methods were applied as prespecified sensitivity analyses to address residual confounding. The PS was estimated using multivariable logistic regression including baseline covariates measured at admission: age, sex, body mass index, onset-to-admission time, baseline National Institutes of Health Stroke Scale score, infarct territory, hypertension, diabetes mellitus, atrial fibrillation, coronary artery disease, previous stroke, and baseline functional measures (Barthel Index, Fugl–Meyer Assessment motor score, and Berg Balance Scale).

Propensity score matching was performed using 1:1 nearest-neighbor matching without replacement with a caliper of 0.2 standard deviations of the logit of the PS. Covariate balance was evaluated using standardized mean differences (SMDs), with SMD <0.10 indicating adequate balance.

Inverse probability of treatment weighting using stabilized weights was also conducted, with weights truncated at the 1st and 99th percentiles to limit extreme values. Complete-case analysis was applied, as no missing data were present in variables used for PS estimation.

**Propensity score diagnostics**

Before matching, several baseline variables showed mild imbalance (maximum SMD = 0.18). After propensity score matching, all baseline covariates achieved good balance (all SMDs <0.10), indicating successful covariate balance between groups. Similar balance was observed after IPTW weighting (all SMDs <0.10).

In the IPTW analysis, stabilized weights showed a mean of 1.00 (SD 0.24), with truncation effectively limiting extreme weights (range 0.42–2.31). The effective sample size after weighting was 164.3, indicating adequate precision of the weighted estimates. Detailed balance diagnostics are presented in Supplementary Tables S4 and S5.

**Supplementary Table S4.** Covariate balance before and after propensity score adjustment

| Covariate | Before matching SMD | After PSM SMD | After IPTW SMD |
| --- | --- | --- | --- |
| Age | 0.08 | 0.03 | 0.02 |
| Male sex | 0.02 | 0.01 | 0.01 |
| BMI | 0.05 | 0.03 | 0.02 |
| Onset-to-admission time | 0.06 | 0.04 | 0.03 |
| Baseline NIHSS | 0.15 | 0.05 | 0.04 |
| Anterior circulation infarct | 0.04 | 0.02 | 0.02 |
| Hypertension | 0.07 | 0.03 | 0.02 |
| Diabetes mellitus | 0.09 | 0.04 | 0.03 |
| Atrial fibrillation | 0.08 | 0.03 | 0.03 |
| Coronary artery disease | 0.07 | 0.04 | 0.03 |
| Previous stroke | 0.1 | 0.05 | 0.04 |
| Baseline BI | 0.12 | 0.06 | 0.05 |
| Baseline FMA-motor | 0.11 | 0.05 | 0.05 |
| Baseline BBS | 0.06 | 0.04 | 0.03 |

Notes: SMD, standardized mean difference; PSM, propensity score matching; IPTW, inverse probability of treatment weighting; BMI, body mass index; NIHSS, National Institutes of Health Stroke Scale; BI, Barthel Index; FMA, Fugl–Meyer Assessment; BBS, Berg Balance Scale. SMD <0.10 indicates adequate balance.

**Supplementary Table S5.** Distribution of IPTW weights

| Statistic | Value |
| --- | --- |
| Mean stabilized weight | 1 |
| Standard deviation | 0.24 |
| Minimum weight | 0.42 |
| Maximum weight | 2.31 |
| Effective sample size | 164.3 |

Supplementary Table S6. Additional analyses of the primary outcome (mRS).

| Analysis | Effect estimate | 95% CI | p value |
| --- | --- | --- | --- |
| Ordinal logistic regression (mRS 0–6) |  |  |  |
| EBEM vs control (common OR) | 2.12 | 1.32–3.41 | 0.002 |
| Logistic regression adjusted for LOS (mRS 0–2) |  |  |  |
| EBEM vs control | 3.21 | 1.44–7.15 | 0.004 |
| Length of stay (per day increase) | 1.03 | 0.97–1.09 | 0.321 |

Notes: mRS, modified Rankin Scale; EBEM, evidence-based early mobilization; OR, odds ratio; CI, confidence interval; LOS, length of stay. Ordinal logistic regression evaluated the shift across the full mRS distribution (0–6). The LOS-adjusted model additionally included length of stay as a covariate in the fully adjusted regression model.
